# Impact of community lifestyle intervention on anthropometric parameters and body composition among overweight and obese women: findings from the MyBFF@home study

**DOI:** 10.1186/s12905-018-0595-z

**Published:** 2018-07-19

**Authors:** Nor Azian Mohd Zaki, Geeta Appannah, Noor Safiza Mohamad Nor, Azahadi Omar, Mansor Fazliana, Rashidah Ambak, Siti Shafiatun Mohsin, Tahir Aris

**Affiliations:** 1 Institute for Public Health, National Institutes of Health, Ministry of Health Malaysia, Kuala Lumpur, Malaysia; 2 Department of Nutrition and Dietetics, Faculty of Medicine and Health Sciences, Universiti Putra Malaysia, Serdang, Selangor Malaysia; 3 Cardiovascular, Diabetes and Nutrition Research Centre, Institute for Medical Research, National Institutes of Health, Ministry of Health Malaysia, Kuala Lumpur, Malaysia; 4 Cheras Health Clinic, Bandar Tun Razak, Kuala Lumpur, Malaysia

**Keywords:** Overweight, Obesity, Low-income women, Community setting, Lifestyle intervention, Malaysia, MyBFF@home study

## Abstract

**Background:**

The prevalence of overweight and obesity among Malaysian women remained high over the past three decades. Collaboration with existing community at-risk may be feasible for wide-scale prevention of overweight and obesity in the country. The aims of this study were to examine the impact of community-based lifestyle intervention among overweight and obese women on their anthropometric and body composition changes as compared to the usual care group.

**Methods:**

This was a quasi-experimental study conducted in low-cost flats in Kuala Lumpur, Malaysia. A total of 255 overweight and obesity individuals aged between 18 to 59 years old were assigned to either the lifestyle intervention group (*n* = 169) or the usual care group (*n* = 146) over a period of 6 months. Individuals in the intervention group received 6 individual lifestyle counselling comprised of physical activity, diet counselling and self-monitoring components aimed to achieve at least 5% weight loss while individuals in the usual care group obtained six sessions of health care seminars from health care providers. These individuals were then followed-up for another 6 months without any intervention as part of maintenance period.

**Results:**

An intention-to-treat analysis of between-groups at 6-month of intervention (β, 95% CI) revealed greater changes in weight among intervention individuals’ (− 1.09 kg vs. -0.99; *p* < 0.018) as compared to the control group. These changes were not sustained during the maintenance phase (between 6 and 12 months). Overall significant improvement at 12th month was found for visceral fat (− 0.78 vs. -0.64; *p*-value = 0.017), although no significant changes between groups were detected either during intervention or maintenance phase (*p* > 0.05). Individuals in the intervention group showed a significant increase for skeletal muscle mass (0.13 kg) than those individuals in the control group (− 0.37 kg), *p* = 0.033, throughout the study period.

**Conclusion:**

This study provides evidence that an overweight and obesity prevention program can be implemented in a community setting, with some reduction of several anthropometric and body composition parameters.

## Background

An increase in the national prevalence of overweight and obesity in Malaysia has been documented over the last three decades [1–7]. Women and of those with low educational attainment were the sub-groups of the population that were particularly affected [5–8]. Similar observations were also reported in cross-sectional and longitudinal studies conducted in developed countries whereby women from disadvantaged background were more likely to be obese compared to those more affluent ones [9–12].

Prevention of overweight and obesity is possible by altering their risk factors. For instance, a weight loss of 7 to 10% of initial body weight (7 to 10 kg) was documented in intensive lifestyle interventions that combined diet, exercise and behavioural approaches after a period of six to 12 months [13, 14]. Although convincing evidence exists, the translation of lifestyle intervention at the community setting is rather uncommon. Lifestyle intervention in the community settings, could provide an effective opportunity to reach a large number of overweight and obese population [15].

Recent evidences have suggested that lifestyle interventions can be conducted in diverse community settings [16–20]. A review by Hillier-Brown et al. concluded that tailored weight loss interventions targeted at overweight and obese individuals who were socio-economically deprived and of those conducted in the community settings showed a general weight reduction up to 2.0 kg over six months of intervention [16]. An intervention study among 106 low-income women in the US concluded a substantial weight loss among individuals in the intervention group compared to individuals in the control group (− 2.0 kg vs. + 0.2 kg; *p* = 0.03) after a six month intervention [17]. Similar finding was also observed in an intervention study conducted among postpartum women living in the deprived areas in the UK [18]. Craigie et al. described that after a 12-week intervention, individuals in the intervention group showed greater reduction in their body weight (− 1.6 kg vs. + 0.2 kg; *p* = 0.018) as well as in BMI (− 0.7 kg m^− 2^ vs 0.1 kg m^− 2^; *P* = 0.009) and body fat (− 1.5 vs. − 0.5; *P* = 0.029) than those in the control group [18]. Another study which consisted of a majority number of women who were at high risk of developing diabetes recorded greater number of significant weight loss in the intervention group (25%) as compared to control group (11%). This outcome was achieved after six months of tailored weight loss programme in the USA [19]. Using an intention-to-treat analysis, a six month randomised controlled trial among a total of 86 low-income African-American observed greater weight loss among individuals in the intervention group than the control after 9 months (− 1.52 kg vs. + 0.61 kg; < 0.01) [20].

Studies on the effectiveness of pragmatic weight loss programmes in community settings from developing countries are limited. While many observational and prevalence studies in Malaysia have assessed the magnitude of overweight and obesity and their related risk factors, to the best of our knowledge, none were on the effectiveness of lifestyle intervention programmes for weight loss among overweight and obese women. Therefore, the aims of this study were to examine the impact of community-based lifestyle intervention among overweight and obese women on their anthropometric and body composition changes as compared to the usual care group.

## Methods

### Study design and participants

My Body Fit and Fabulous (MyBFF@home) study was a quasi-experimental study conducted in 14 low cost flats in the Federal Territory of Kuala Lumpur (Klang Valley). The recruited individuals were overweight and obese housewives aged 18–59 years old. Inclusion criteria were including those homestay housewives who were not working for at least six months and have no jobs or fixed income. Prior to the study, all eligible individuals were given written informed consent.

Study individuals were assigned into two groups (intervention and control groups) and were assessed for a period of 12 months. During the first 6 months, individuals in the intervention group received the MyBFF@home package of combined dietary counselling and modification, exercise and physical activity programme as well as self-monitoring tools; while the control group attended group seminar and discussion on women’s health during the follow-up sessions. During weight maintenance phase (7th through 12th month), no intervention activities were carried out and both groups were followed up during this period. Detailed methodology of the study was described by Mohammad Nor et al. [21].

### Anthropometry variables

**Fig. 1:**
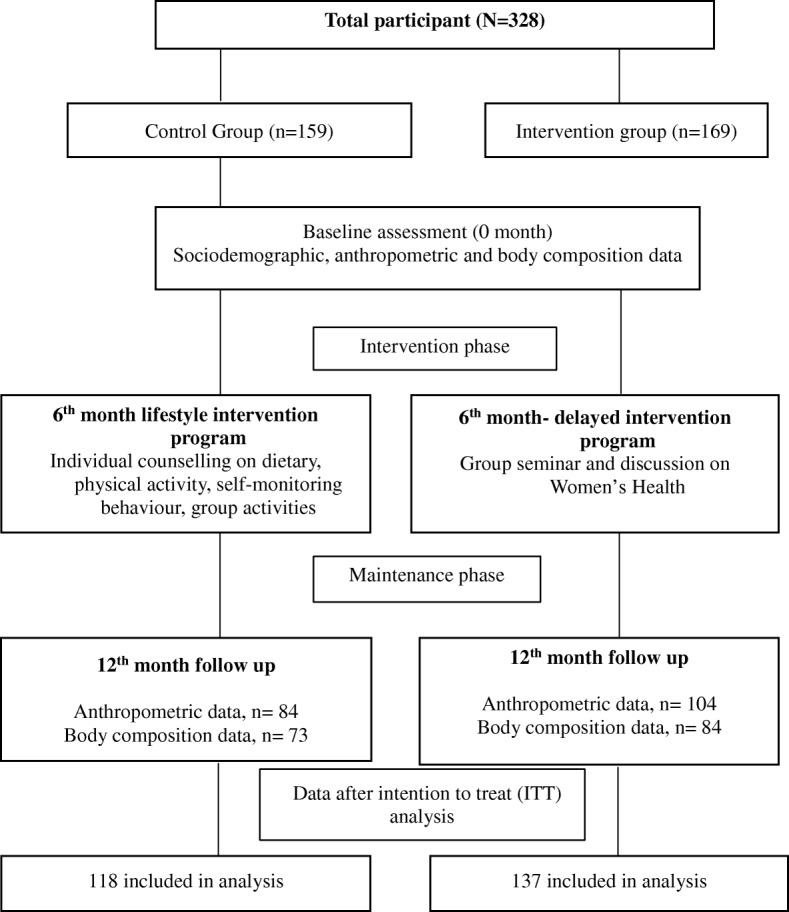
Flow of individuals completed anthropometry and body composition assessments

Assessments of anthropometry that included body weight, height, waist and hip circumference were performed five times in total (baseline, visits 1, 2, 3 and 4) during the intervention phase. During the maintenance phase, anthropometric assessments were measured during the 9th and 12th month (visits 5 and 6). The anthropometric measurements were conducted according to a standard protocol described previously [22]. The flow of study individuals completing their anthropometry and body composition assessments is shown in Fig. 1. Body mass index (BMI) classification cut-off points from the World Health Organization (WHO) guidelines was used to define overweight (≥25 to 29.9 kg/m^2^) and obese (≥30 kg/m^2^) [23]. Only individuals with BMI of 25.0 to 39.9 kg/m2 were included in this study. All measurements were taken twice and mean values of measurements were used for analysis.

### Body composition variables

Body composition assessments including body fat mass, body fat percentage, visceral fat and muscle mass were performed three times over the study period (baseline, 6th and 12th month follow-up). As previously described, individuals in this study were assessed for their body composition by using bioelectrical impedance analyser (BIA) (In Body 720) [21]. BIA was used as the tool was simple to use and non-invasive although it was quite heavy. Study by Ling et al. had showed that BIA gives consistent and accurate estimation of body composition measurements and less intra- and inter-observer variability [24]. Studies had been conducted to validated these tools to measure body composition [25, 26].

### Socio-demographic variables

Socio-demographic profiles including age, ethnicity, education level, marital status, household income and number of children were obtained during the baseline assessment.

### Statistical analysis

A total of 328 individuals were recruited at the baseline (169 individuals in intervention group and 159 individuals in control group). However, only 255 individuals had completed all data at baseline. Multiple imputation method via regression analysis was done to handle the missing values in anthropometry and body composition data. An intention to treat (ITT) analysis comprised a total of 137 individuals in the intervention and 118 individuals in the control group at all follow-ups sessions.

In this study, a generalised estimating equation (GEE) analysis was performed to take into account of the potential correlations of data between the follow-ups [27]. An “auto-regressive’ working correlation structure was specified for the GEE models in the context of balanced prospective data as measurements that are collected closer in time are more likely to be highly correlated compared to measurements further apart in time. Balanced data occurs when individuals were assessed literally at the same intervals, a typical occasion of intervention studies.

The independent variables in GEE analysis included variables during all visits (baseline to 6th month -intervention phase, 6th to 12th month -maintenance phase and baseline to 12th month -overall) as well as covariates such as age (continuous variable) and education level (categorical variable). Dependent variables were individual anthropometric and body composition parameters; e.g. weight, BMI, waist and hip circumference, waist-hip ratio, fat mass density, percentage of body fat, visceral fat and skeletal muscle mass. The analysis was repeated separately for each outcome and different phases of the study i.e. intervention (baseline, visits 1, 2, 3 and 4), maintenance (visits 4, 5 and 6) and overall visit (baseline, visits 1, 2, 3, 4, 5 and 6). The effect of intervention on separate anthropometric measurements was using linear regression models.

Since GEE requires variables collected at least three times or more, the analysis could not be performed for body composition parameters for intervention and maintenance phase. The mean differences between the sixth month and baseline for the intervention phase and 12th and 6th month for maintenance phase were tested using t-test. Overall changes of the body composition parameters included data collected at baseline, 6th and 12th month (3 times). As such, GEE analysis was performed and adjusted for covariates. Intervention effects on the body composition parameters for overall changes were tested using linear regression models. Analyses were conducted using Stata 14.0 (StataCorp LP, College Station, TX).

## Results

The descriptive characteristics of the individuals at baseline are as reported by Noor Safiza et al. [21]. Table 1 describes the summary of anthropometric and body composition measurements at each follow-up. Baseline body weight in the intervention group (75.9 ± 11.3 kg) was significantly higher than the control group (72.6 ± 11.5 kg). Mean ± SD for skeletal muscle mass during baseline was higher for individuals in the intervention groups (22.2 ± 2.8 kg) compared to those in the control group (21.5 ± 3.0 kg).

**Table 1:** Summary characteristics of anthropometric and body composition measurements

Outcome variable	Mean (SD)						
	Baseline	Month 1	Month 2	Month 3	Month 6	Month 9	Month 12
Weight (kg)							
Intervention Group (*n* = 137)	75.9* (11.3)	75.9* (11.4)	75.8* (11.3)	75.5* (11.2)	74.9* (11.5)	75.7 (11.5)	75.4* (11.4)
Control Group (*n* = 118)	72.6 (11.5)	72.5 (11.3)	72.3 (11.5)	72.2 (11.5)	71.7 (11.3)	72.5 (11.8)	72.0 (12.1)
BMI (km/m^2^)							
Intervention Group (n = 137)	31.6 (4.1)	31.6* (4.2)	31.3 (4.1)	31.2 (4.2)	31.1 (4.2)	31.4 (4.2)	31.4 (4.3)
Control Group (*n* = 118)	30.9 (4.2)	30.4 (3.9)	30.9 (4.3)	30.36 (4.1)	30.3 (4.2)	30.8 (4.3)	30.6 (4.5)
Waist circumference (cm)							
Intervention Group (*n* = 137)	94.8 (10.1)	93.8 (10.0)	93.2 (9.8)	92.71 (9.6)	91.8 (9.2)	91.5 (9.6)	90.7 (9.0)
Control Group (n = 118)	92.8 (9.4)	92.5 (8.9)	91.8 (9.0)	90.72 (9.5)	90.5 (9.6)	89.2 (9.9)	88.8 (9.2)
Hip circumference (cm)							
Intervention Group (n = 137)	111.4 (9.2)	110.5 (9.4)	109.5 (9.2)	108.8 (8.9)	109.0* (8.9)	109.8 (9.4)	110.8* (9.4)
Control Group (*n* = 118)	109.5 (8.7)	108.8 (8.8)	107.5 (9.2)	107.2 (8.7)	106.7 (9.1)	107.8 (9.8)	107.1 (9.8)
Waist-hip-ratio							
Intervention Group (n = 137)	0.85 (0.1)	0.85 (0.1)	0.85 (0.1)	0.85 (0.1)	0.84 (0.1)	0.83 (0.1)	0.82 (0.1)
Control Group (n = 118)	0.85 (0.1)	0.85 (0.1)	0.85 (0.1)	0.85 (0.1)	0.84 (0.1)	0.83 (0.1)	0.82 (0.1)
Fat mass (kg)							
Intervention Group (n = 137)	34.8 (7.9)	–	–	–	33.6 (8.1)	–	33.9 (9.2)
Control Group (n = 118)	32.8 (7.9)	–	–	–	31.8 (8.1)	–	32.8 (9.2)
Percentage Body fat (%)							
Intervention Group (n = 137)	45.2 (5.0)	–	–	–	43.8 (5.4)	–	44.5 (7.4)
Control Group (n = 118)	44.7 (4.7)	–	–	–	43.9 (5.8)	–	45.1 (7.3)
Visceral fat area (cm^2^)							
Intervention Group (n = 137)	128.1* (23.9)	–	–	–	122.5 (23.3)	–	124.8 (26.9)
Control Group (*n* = 118)	120.5 (23.9)	–	–	–	117.2 (24.2)	–	118.5 (27.7)
Skeletal muscle mass (kg)							
Intervention Group (*n* = 137)	22.2* (2.8)	–	–	–	22.2* (2.3)	–	22.4* (3.0)
Control Group (n = 118)	21.5 (3.0)	–	–	–	21.3 (3.0)	–	21.1 (3.0)

Value are presented as mean ± standard deviation

*P < 0.05 for independent T- test

A greater number of individuals in the intervention group lost more weight (> 2 to 20 kg) than those in the control group (45% vs. 41%; *p* = 0.09) over a period of six months (Table 2).

**Table 2:** Summary of weight loss over the intervention and maintenance phase

Weight change category, n (%)	Intervention phase (Baseline - 6 month)			Maintenance phase (6 month - 12 month)		
	Control	Intervention	*p-value	Control	Intervention	*p-value
Gained > 2%	17 (14.5)	19 (13.9)	0.09	39 (33.3)	55 (40.1)	0.20
Maintained +/− 2%	52 (44.4)	57 (41.6)		46 (39.3)	57 (41.6)	
Loss > 2–5%	28 (23.9)	49 (35.8)		15 (12.8)	16 (11.7)	
Loss > 5–20%	20 (17.1)	12 (8.8)		17 (14.5)	9 (6.6)	
Total	117 (100)	137 (100)		117 (100)	137 (100)	

Value are presented as mean ± standard deviation

*P<0.05 for Independent T-test

### Changes in anthropometric and body composition measurements

#### Overall

Overall changes of weight between baseline and 12th month was found to be significant for both the groups (Table 3). However, the changes between the groups indicated a greater significant weight reduction in the control compared to intervention group (− 0.1 kg vs. 0.1 kg; *p* = 0.013). No significant overall changes were observed for BMI, waist and hip circumference as well as waist-hip ratio (Table 3).

**Table 3:** Changes in the anthropometric parameters during intervention and maintenance phases

Anthropometric parameters	Intervention phase (0–6 months)			Group Effect (p-value)	Maintenance phase (6–12 months)			Group Effect (*p*-value)	Overall (0–12 months)			Group Effect (p-value)
	β	95% CI	p-value		β	95% CI	p-value		β	95% CI	p-value	
Weight (kg)												
Intervention Group (*n* = 137)	−1.04	− 1.34, − 0.74	< 0.001	0.013	− 0.45	− 0.93, 0.27	0.065	0.013	− 0.10	− 0.15, − 0.05	< 0.001	0.013
Control Group (n = 118)	− 0.89	− 1.26, − 0.53	< 0.001		− 0.65	− 1.47, 0.17	0.122		− 0.11	− 0.19, − 0.03	0.006	
BMI (km/m^2^)												
Intervention Group (*n* = 137)	− 0.49	− 0.63, − 0.34	< 0.001	0.190	− 0.21	− 0.26, − 0.15	< 0.001	0.200	− 0.05	− 0.07, − 0.03	< 0.001	0.195
Control Group (n = 118)	− 0.39	− 0.58, − 0.21	< 0.001		−0.27	− 0.43, − 0.12	< 0.001		− 0.04	− 0.07, − 0.02	< 0.001	
Waist circumference (cm)												
Intervention Group (n = 137)	−3.05	− 3.82, − 2.28	< 0.001	0.101	− 3.86	− 4.83, − 2.89	< 0.001	0.096	−0.64	− 0.8, − 0.5	< 0.001	0.098
Control Group (n = 118)	− 2.34	− 3.28, − 1.40	< 0.001		− 3.84	−4.90, − 2.77	< 0.001		− 0.64	− 0.8, − 0.5	< 0.001	
Hip circumference (cm)												
Intervention Group (n = 137)	−2.39	− 2.97, − 1.81	< 0.001	0.070	−0.92	−1.49, − 0.36	0.001	0.076	− 0.21	− 0.3, − 0.1	< 0.001	0.072
Control Group (n = 118)	−2.69	−3.35, − 2.02	< 0.001		− 2.09	− 2.85, − 1.34	< 0.001		− 0.41	− 0.5, − 0.3	< 0.001	
Waist-hip-ratio												
Intervention Group (n = 137)	− 0.010	− 0.018, − 0.003	0.008	0.896	− 0.029	− 0.03, − 0.02	< 0.001	0.893	− 0.004	−0.005, − 0.003	< 0.001	0.897
Control Group (n = 118)	−0.005	− 0.013, 0.003	0.284		−0.019	− 0.04, − 0.02	< 0.001		− 0.003	−0.004, − 0.002	< 0.001	

All models were adjusted for age and education level

A greater overall reduction was observed for fat mass density (− 0.8 kg vs. -0.02 kg; *p* = 0.053), body fat percentage (− 0.66% vs. 0.35%; *p* = 0.532) and visceral fat (− 3.27 cm^2^ vs. -2.04 cm^2^; *p* = 0.016) in the intervention group compared to control group (Table 4). Individuals in the intervention group showed a significant increase for skeletal muscle mass (0.13 kg) than those individuals in the control group (− 0.37 kg), *p* = 0.033.

**Table 4:** Changes in the body composition parameters during intervention and maintenance phases

Body composition parameters	Intervention phase (0–6 months)			Group Effect (p-value)	Maintenance phase (6–12 months)			Group Effect (p-value)	Overall (0–12 months)			Group Effect (p-value)
	Baseline mean (SD)	6 months mean (SD)	Mean Differences (95%CI)		6 months mean (SD)	12 months mean (SD)	Mean Differences (95%CI)		β coefficient	95%CI	p-value	
Fat mass (kg)												
Intervention Group (n = 137)	34.8 (7.9)	33.6 (8.1)	−1.19 (− 1.70, −0.68)	0.592	33.6 (8.1)	33.9 (9.2)	0.38 (−0.52, 1.27)	0.346	−0.82	−1.56, − 0.08	0.002	0.053
Control Group (*n* = 116)	32.8 (7.9)	31.8 (8.1)	−0.98 (−1.56, − 0.41)		31.8 (8.1)	32.8 (9.2)	1.0 (0.08, 1.89)		−0.02	−0.82, 0.78	0.953	
Body fat (%)												
Intervention Group (n = 137)	45.2 (5.0)	43.8 (5.4)	−1.40 (−2.12, −0.67)	0.274	43.8 (5.4)	44.5 (7.4)	0.74 (−0.31, 1.79)	0.547	−0.66	−1.56, 0.24	0.152	0.532
Control Group (n = 116)	44.7 (4.7)	43.9 (5.8)	−0.85 (−1.50, − 0.20)		43.9 (5.8)	45.1 (7.3)	1.21 (0.07, 2.36)		0.35	−0.62, 1.32	0.482	
Visceral fat area (cm^2^)												
Intervention Group (n = 137)	128.1 (23.5)	122.5 (23.3)	−5.56 (−7.38, −3.74)	0.113	122.5 (23.3)	124.8 (26.9)	2.30 (−0.38, 5.0)	0.614	−3.27	−5.48, −1.05	0.004	0.016
Control Group (n = 116)	120.5 (23.9)	117.2 (24.2)	−3.37 (−5.53, −1.20)		117.2 (24.2)	118.5 (27.7)	1.35 (−1.20, 3.89)		−2.04	−4.49, 0.39	0.011	
Skeletal muscle mass (kg)												
Intervention Group (n = 137)	22.2 (2.8)	22.2 (2.3)	−0.05 (−0.30, 0.21)	0.324	22.2 (23.0)	22.4 (3.0)	0.17 (−0.16, 0.50)	0.189	0.13	−0.18, 0.44	0.418	0.033
Control Group (n = 116)	21.5 (3.0)	21.3 (3.0)	−0.21 (− 0.43, 0.001)		21.3 (3.0)	21.1 (3.0)	−0.15 (− 0.51, 0.21)		− 0.37	−0.68, − 0.06	0.020	

Overall models were adjusted for age and education level

#### Intervention phase

A slightly higher changes of weight loss was observed in the intervention group compared to the control group (− 1.0 kg vs. -0.9 kg; *p*-value = 0.013). BMI and waist circumference also demonstrated significant greater changes in the intervention group compared to the control group over the 6 months of intervention (within group) but the changes were not different between the groups (Table 3). Hip circumference and waist-hip ratio, except for the control group showed significant changes from baseline but the changes were comparable between the groups (*p* > 0.05).

Table 4 shows that greater changes between 6-month measurement and baseline were observed for fat mass density (− 1.2 kg vs. -1.0 kg), body fat percentage (− 1.40% vs. -0.85%) and visceral fat (− 5.56 cm^2^ vs. -3.37 cm^2^) in the intervention group as compared to control group; however, the changes between the groups were not significant (*p* > 0.05). On the other hand, skeletal muscle mass showed higher changes in the control group compared to intervention group (− 0.2 kg vs. -0.1 kg; *p* = 0.324).

#### Maintenance phase

Table 3 describes changes in weight, BMI and hip circumference during maintenance phase that were favoured by control group compared to intervention group; however, the changes were only significant for weight (− 0.7 kg vs. -0.5 kg; *p* = 0.013). Significant higher changes in waist circumference (− 3.86 cm vs. -3.84 cm) and waist-hip ratio (− 0.029 vs. -0.01) were presented in the intervention group compared to the control group over the duration of maintenance phase, but the changes were not different between the groups (*p* > 0.05).

In contrast to the intervention phase (baseline to month 6), Table 4 describes no reduction in the body composition parameters during maintenance phase in both groups (except for skeletal muscle mass). Greater gains were observed in the control group compared to intervention group for fat mass density (1.0 kg vs. 0.4 kg) and body fat percentage (1.21% vs. 0.74%) but the gains were not different between the groups (p > 0.05). Nevertheless, individuals in the intervention groups recorded greater gain in the visceral fat than those in the control group (2.30 cm^2^ vs. 1.35 cm^2^; *p* = 0.614). Greater gains in skeletal muscle mass was found for individuals in the intervention group (0.2 kg), whereas individuals in the control group lost an average of 0.2 kg during this phase (*p* = 0.189).

## Discussion

The current study suggests that a short-term lifestyle intervention over a period of six months in community settings can yield small weight loss compared with standard care. Furthermore, individuals in the intervention group demonstrated a significant overall improvement in fat mass and visceral fat and increased skeletal muscle mass over 12 months of the study. In addition, a greater proportion of the individuals in the intervention group loss more weight than those in the control group.

Although the weight loss over the period of six months (intervention phase) was statistically significant between the groups, it is crucial to discuss the clinical implications of such findings. It was noted that on the average, individuals in the intervention phase lost 1.0 kg and reduced approximately 1.3% of their body weight. Though trivial, a small reduction in body weight (5%) has been related to improvements on obesity-related health outcomes such as high blood pressure and diabetes [28]. Furthermore, the deterrence of weight gain (without any weight loss), may be useful, as some individuals incline to experience weight gain over time in the absence of any intervention [29]. The small percentage of weight loss could be due to the fact that individuals in the intervention group gained skeletal muscle mass over the 12 months of follow-up. The modest amounts of physical activity (dumb-bell exercise) as well as energy restrictions in the intervention group could have attenuated the loss of muscle mass and this finding was in line with those reported by Chomentowski et al. in 2009, Yoshimura et al. in 2014 and Kim et al. in 2017 [30–32]. Conversely, weight loss due to diet alone was reported to cause an undesirable reduction in skeletal muscle mass, which is, more likely to decrease muscle strength and physical performance [31, 33, 34].

The weight loss result found in this study was in contrast with other studies targeting women whereby a reduction of at least 2.0 kg was reported [17, 19]. A more intensive intervention may be useful in order to attain significant amount of weight loss for Malaysian women. Incorporating behavioural component into the intervention programme may be important for weight loss, or frequent follow-up may be useful for a longer term of weight loss maintenance and improving obesity-related health outcomes. This was indicated in studies where long term intensive behavioural lifestyle modifications were applied whereby a greater proportion of individuals achieved clinically significant weight loss (> 5%) [35, 36]. However, the applicability and sustainability of such weight loss is yet to be proven, as they are usually very time intensive and suitable for highly motivated individuals of the population.

While the baseline mean BMI of the individuals in the intervention and control groups were 31.4 kg/m^2^ and 30.9 kg/m^2^ respectively, approximately 19% of the individuals in both groups were found to have BMI greater than 35 kg/m^2^ [21]. While a few studies have suggested significant diet-induced weight loss in severely obese individuals, it should be also pointed out that many other lifestyle intervention studies among severely obese were not responded well, particularly having an issue with low retention rates [37–39]. On the other hand, studies that have incorporated intensive medical weight loss programs at the primary care level showed better weight loss outcomes compared to the usual care [37, 40, 41].

The group difference for weight loss was no longer favourable for intervention group at 12th month, in this study. At 12th month, more individuals in the intervention group regained their weight loss compared to those subjects in the control group (32% vs. 19%). A weight loss of at least 5% was achieved and maintained for 12 months by a small number of individuals (12.4%) in the intervention group. Nevertheless, it is of interest that individuals in the control group also showed improvements in all-anthropometric and body composition parameters to those in the intervention group. It is likely that the individuals in the control group also changed their obesity-related behaviour and some other parameters during the study. As the recruitment of individuals in both groups was conducted in the city of Kuala Lumpur, it might be that individuals from the intervention group made contact with the controls or vice-versa and this could have influenced the behaviour change among the individuals in the control group. Through these contacts, it could be that individuals in the intervention groups have shared the advice, educational content and encouragement they received with those in the control group and subsequently affected the weight-loss behaviour in individuals from the control group. Secondly, since all individuals were evaluated for anthropometric, dietary, body composition, physical activity, biological and other lifestyle parameters, these might have influenced behaviour changes among individuals in the control group, independent of the study group allocation. Thirdly, the likelihood of the ‘Hawthorne effect’ could have an effect on the improvements experienced by the individuals in the control group. This effect may be demarcated as the unpredicted and sometimes as an unexplained response to intervention in individuals who are aware of study participation [42].

The overall reduction of visceral adiposity, particularly, among the individuals in the intervention compared to those in the control group might be due to the reported weight loss. As the intervention program in this study emphasised both energy restrictions and physical activity, the reduction in visceral adiposity maybe explained by a lipolysis process [43]. During lipolysis (usually when exercising), triglycerides stored in visceral fat depot are broken down and free fatty acids are released. Increased physical activity, particularly has been documented to be associated with greater sympathetic nervous system activity and visceral fat has a larger lipolytic potential than subcutaneous fat and therefore, it is not surprising that visceral fat may be reduced when physical activity is part of the intervention [44]. On the contrary, Ross et al. suggested that a similar weight loss through physical activity or calorie restriction component alone might also decrease abdominal visceral fat [45]. Therefore, the magnitude of visceral fat reduction is influenced mostly through the degree of weight loss.

There were few limitations of this study worth mentioning. First, the incorporation of a quasi-experimental study in community settings hindered the randomisation effect and might not have sufficiently controlled the possibility of confounding factors. However, we made sure that baseline characteristics of the measured parameters were comparable between the intervention and control groups [21]. Despite the similarity found between the groups, we attempted to adjust for covariates such as age and education level, in all the GEE models. Next, the use of regression method in the intention-to-treat analysis may have obscured the true outcomes. However, we had performed a sensitivity analysis, whereby a separate analysis using complete cases was performed (data not shown). The results among those complete cases suggested similar findings to those shown in this study, indicating that loss to follow-up was probably not a bias in this study. As mentioned earlier, the possibility of contamination might have occurred between the intervention and control groups as the radius of the study locations were just under 30 km. The absence of objectively collected dietary intake and physical activity data was another limitation in this study. However, most studies that have used questionnaires for dietary and physical activity assessments reported good reliability [46, 47]. It could be that we failed to detect any differences between the groups for most of the anthropometric and body composition parameters (except for weight during intervention phase) due to power of the study being inadequate. We had to remove a total of 32 and 42 respondents in the intervention and control groups respectively, as they did not provide baseline data (absent on the day of baseline data collection). However, the strengths of our study were based on the repeated measures and the use of widely acknowledged lifestyle questionnaires. To the very best of our knowledge, this is the first study to focus on lifestyle intervention among women in the community settings in Malaysia. Another strength of this study including the translational potential as it could be delivered to a group of women in somewhat challenging setting of a low socio-economic community.

## Conclusion

In the context of low socio-economic communities, this study suggests that a short-term lifestyle intervention could produce a small percentage of weight loss and decreased visceral fat after six and 12 months respectively, and an overall improvement in the skeletal muscle mass over 12 months of the study. The addition of behavioural component to the intervention package and longer-term trials may support more weight loss and improvements in body composition parameters.

## Abbreviations

BIA: Bioelectrical impedance analyser
BMI: Body mass index
GEE: generalised estimating equation
ITT: intention to treat
MyBFF: My Body is Fit and Fabulous
